# Endoscopic ultrasound-guided gastrojejunopexy using an improvised T-anchor for successful endoscopic gastrojejunostomy

**DOI:** 10.1055/a-2208-5132

**Published:** 2023-12-11

**Authors:** Sanjay M. Salgado, Kamal Maher Hassan, Mohamad-Noor Abu-Hammour, David L. Carr-Locke, Kartik Sampath, Reem Z. Sharaiha, SriHari Mahadev

**Affiliations:** 1 Gastroenterology and Hepatology, Weill Cornell Medicine/New York-Presbyterian Hospital, New York, United States

Endoscopic ultrasound-guided gastrojejunostomy (EUS-GJ) is a minimally invasive alternative treatment for gastric and duodenal outlet obstruction. However, technical difficulty arises from bowel motility. To address this, we present a novel technique using an improvised EUS-directed T-anchor to improve safety and feasibility, particularly for patients with ascites or retained intragastric food.

**Fig. 1:**
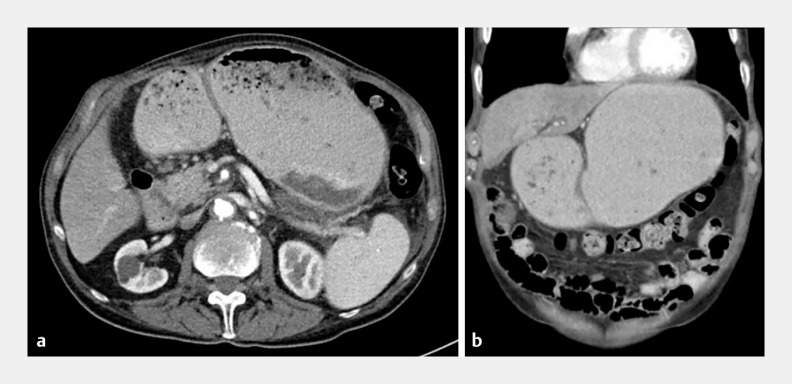
Abdominal computed tomography scan was consistent with gastric outlet obstruction.

**Fig. 2:**
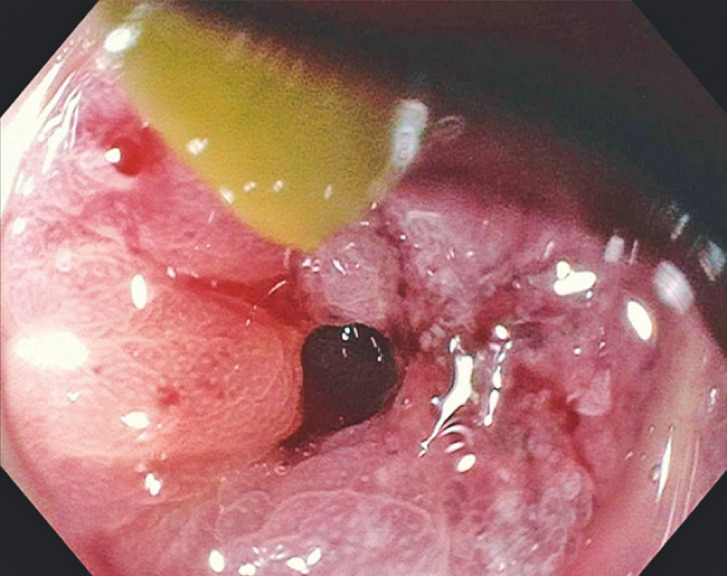
Endoscopic evaluation confirming severe gastric narrowing.

**Fig. 3:**
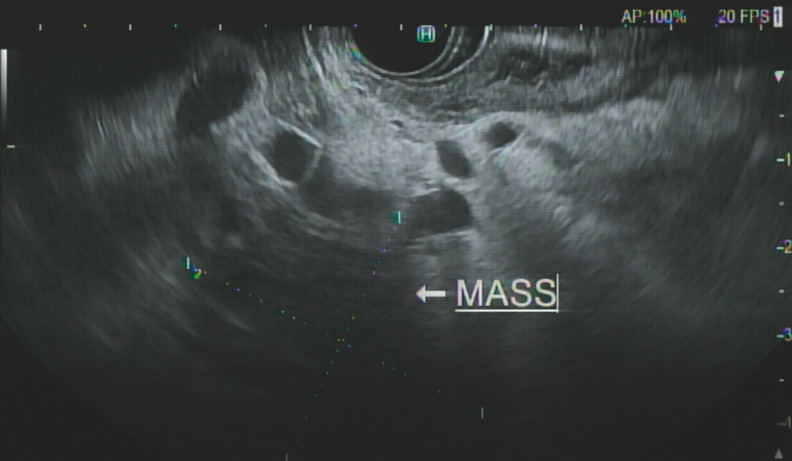
Endoscopic ultrasound demonstrating a large pancreatic mass.

A 92-year-old man developed gastric outlet obstruction due to a pancreatic head mass ( Fig. 1 , Fig. 2 , Fig. 3 ). Through a 10-Fr catheter, the jejunum was distended with methylene blue, contrast, and saline. The stylet of a 19-gauge EUS needle was withdrawn, and the tip of the needle was preloaded with an endoscopic suture acting as a T-fastener ( Video 1 ). The needle and suture were then passed through the working channel of the echoendoscope and punctured the gastric wall into the target jejunal loop. The stylet of the needle was then advanced such that the suture was released into the jejunal lumen. The needle was then withdrawn, leaving the suture in place, and an endoscopic suture cinch was deployed, affixing the stomach and jejunal walls. The 19-gauge EUS needle was then reintroduced, and the same loop of jejunum was punctured. A guidewire was then passed into the jejunum and coiled, without concern of jejunal migration. Finally, a 15-mm electrocautery-enhanced lumen-apposing metal stent (LAMS) was deployed over the wire.

Endoscopic ultrasound-guided (EUS) gastrojejunostomy using an improvised EUS-directed T-anchor in a patient with severe gastric outlet obstruction due to a pancreatic head mass.Video 1

Endoscopic gastrojejunopexy offers the potential to make EUS-GJ a safer and more reliable procedure by preventing jejunal migration away from the gastric wall and permitting the use of a guidewire to ensure intraluminal placement of the LAMS. Without a dedicated EUS-directed T-anchor system, an endoscopic suture and cinch can be improvised to affix the jejunum to the gastric wall to accomplish this maneuver.

Gastrojejunopexy-assisted endoscopic gastrojejunostomy may potentially increase the safety and feasibility of the endoscopic gastrojejunostomy, by minimizing the risk of jejunal migration. Dedicated EUS-directed T-anchors are needed.

Endoscopy_UCTN_Code_TTT_1AS_2AB

